# Plasma levels of the proangiogenic protein CXCL16 remains elevated for 1 month after minimally invasive colorectal cancer resection

**DOI:** 10.1186/s12957-018-1418-2

**Published:** 2018-07-07

**Authors:** H. M. C. Shantha Kumara, Erica Pettke, Abhinit Shah, Xiaohong Yan, Vesna Cekic, Melissa Alvarez Downing, Nipa Dilip Gandhi, Richard L. Whelan

**Affiliations:** 1 Division of Colon and Rectal Surgery, Department of Surgery, Mount Sinai West Hospital, Suite 7B, 425 West, 59th Street, New York, NY 10019 USA; 2 Icahn School of Medicine at Mount Sinai, New York, NY 10029 USA

**Keywords:** Minimally invasive colorectal cancer resection, CXCL16

## Abstract

**Background:**

Inflammation-induced endothelial precursor cell recruitment and angiogenesis are thought to be associated with CXCL16-CXCR6 pair activity. This study’s main purpose was to determine plasma CXCL16 levels after minimally invasive colorectal resection (MICR) for colorectal cancer (CRC); an adjunct study assessed wound fluid (WF) and plasma CXCL16 levels in a separate group of CRC patients.

**Methods:**

CRC patients who had MICR and for whom plasma was available in a tissue bank were eligible. Plasma samples were collected preoperatively from all patients. Samples were also collected on postoperative days (POD) 1 and 3 and at various late postoperative time points (POD 7–34). In a separate study, blood and intra-abdominal wound fluid (WF) samples were collected from CRC MICR patients (pts). Samples were stored at − 80 °C. CXCL16 levels were determined via ELISA. The Wilcoxon signed-rank and Mann and Whitney tests were used for analysis.

**Results:**

*Main study*: 86 CRC pts. were included. The mean preoperative plasma CXCL16 level was 2.36 ± 0.57 ng/ml. Elevated mean plasma levels (*p* <  0.0001 × first 4 time points) were noted on POD 1 (2.82 ± 0.81, *n* = 86), POD 3 (3.12 ± 0.77, *n* = 82), POD 7–13 (3.28 ± 0.88, *n* = 64), POD 14–20 (3.03 ± 0.62, *n* = 24), POD 21–27 (3.06 ± 0.67, *n* = 20, *p* = 0.0003), and POD 28–34 (3.17 ± 0.43, *n* = 11, *p* = 0.001) vs. preop levels. *WF study*: In the adjunct study, plasma and WF CXCL16 levels were determined for 23 CRC MICR pts. WF levels at all time points were significantly elevated over plasma levels.

**Conclusion:**

Plasma CXCL16 levels were elevated for 4 weeks after minimally invasive colorectal resection for cancer. Also, WF CXCL16 levels were 3–10 times greater than the corresponding plasma concentrations. The source of the late plasma elevations may be the healing wound. Increased plasma CXCL16 levels may promote tumor angiogenesis in the first month after MICR.

## Background

In the USA, colorectal cancer (CRC) is one of the most commonly diagnosed cancer types; 135,430 new cases and 50,260 deaths are expected in 2017 [1]. Surgical resection remains the main stay of treatment for patients with stage I–III disease [1]. Despite surgery and, where indicated chemo- and radiotherapy, recurrence develops in a substantial percentage of patients.

Interestingly, there is both human and experimental evidence that colon resection-related surgical trauma and surgical trauma in general are linked to tumor establishment and tumor growth after [2–6]. Thus, the surgery necessary to remove the tumor may stimulate the growth of residual micro-metastases. In the murine setting, the use of laparoscopic methods, vs. open technique, is associated with improved oncologic outcomes [7–10]. In humans, however, no differences in long-term survival or recurrence rates have been demonstrated in randomized trials wherein minimally invasive surgery (MIS) and open colectomy methods were compared [11, 12]. Proposed mechanisms leading to stimulated tumor growth and metastasis after surgery include immunosuppression, tissue trauma-associated adrenergic response, and the wound bed environment that can directly illicit an inflammatory cascade linked to tumor initiation, invasion, and ultimately metastasis [2–4, 13–15]. A critical component necessary for tumor growth and invasion beyond 2 mm is angiogenesis [16]. Interestingly, there is an enlarging body of evidence that colorectal resection in humans is linked to changes in plasma composition that render it pro-angiogenic for 3–5 weeks after surgery.

Plasma levels of the following proteins have been shown to be significantly and persistently elevated after minimally invasive colorectal resection (MICR): angiopoietin-2 (Ang-2), soluble vascular cell adhesion molecule-1 (sVCAM-1), vascular endothelial growth factor (VEGF), placental growth factor (PlGF), monocyte chemotactic protein-1 (MCP-1), chitinase-3-like protein 1 (Chi3L1), interleukin-8 (IL8), and matrix metalloproteinase-3 (MMP-3) among others [17–23]. Further, it has been demonstrated that plasma from the second and third postoperative weeks after colorectal resection stimulates endothelial cell (EC) proliferation, migration, and invasion in in vitro cultures when compared to results obtained with preoperative plasma [24, 25]. These proangiogenic proteins in the plasma may stimulate tumor angiogenesis in patients with residual tumor tissue after minimally invasive colorectal resection. The etiology and source of the additional protein in the circulation has not been well determined; however, there is some evidence that the healing wounds may be a source since VEGF levels in wound fluid have been noted to be elevated well beyond blood levels [26–28].

Transmembrane chemokine CXCL16 is found on leukocytes, endothelial cells, and other tissues that stimulate EC proliferation and chemotaxis. A soluble CXCL16 shed from the membrane likely accounts for its activity. CXCR6 is the receptor found on cells at sites of inflammation. Endothelial precursor cell recruitment and angiogenesis induced by pro-inflammatory stimuli are thought to be associated with CXCL16 and CXCR6 pair activity [29–34]. Specifically, CXCL16 and CXCR6 have been shown to play a role in the epithelial-mesenchymal transition that is thought to initiate EC proliferation, migration, and invasion [35, 36]. Expression of CXCL16 is reported in many cancers including CRC [35–38]. The impact of surgery on blood levels of CXCL16 is unknown. This study’s purpose was twofold: first, to determine plasma CXCL16 levels in CRC patients before and during the first 5 weeks after MICR (study A) and secondly, in a separate smaller study of CRC patients, to determine and compare plasma and wound fluid levels (study B).

## Methods

### Study population

Consenting patients with colorectal malignancies who underwent MICR, and had sufficient plasma available, were included in study A. All patients had previously been enrolled in an Institutional Review Board (IRB) approved tissue and data collection protocol (IRB of the Mount Sinai Icahn School of Medicine, New York; IRB reference number:GCO#1: 16-2619). IRB-approved (IRB of the Mount Sinai Icahn School of Medicine, New York; IRB reference NO: GCO#16-2619) informed consent was obtained from all participants. The purpose of this IRB protocol was to determine the physiologic and immune-oncologic impact of major abdominal surgery. As regards study B, CRC patients who met the following criteria were eligible: had enrolled in the abovementioned tissue and data collection protocol, underwent MICR during which a Jackson-Pratt drain was placed in the pelvis (at the surgeon’s discretion), agreed postoperatively to enter the IRB-approved wound fluid study (IRB of the Mount Sinai Icahn School of Medicine, New York; IRB reference NO: GCO#1: 16-1863), and IRB-approved (Institutional Review Board of the Mount Sinai School of Medicine, New York NY; IRB reference NO: GCO#16-1863) informed consent for participation in the study was obtained from all participants. Adult subjects (age 18 or older) who plan to undergo MICR at Mount Sinai West Hospital would be eligible to enroll in this research study. Enrolled patients did not receive a novel drug, or other therapy, in the perioperative period. Patients who had perioperative blood transfusion were excluded. Likewise, immunosuppressed patients and patients who underwent emergent operations were also excluded. The demographic, operative, clinical, and early outcome data were collected from the prospective database, as well as hospital and office charts.

### Blood sampling and processing

For a CRC patient to be eligible for inclusion in study A, a preoperative and at least three postoperative plasma samples, including one specimen from POD 7 or later, had to be available. Of note, after hospital discharge, blood samples were obtained at the time of follow-up office visits that were not scheduled on specific PODs. Also, many patients refused to give blood at late time points. As a result, the number of blood samples available for a given late postoperative day was small. Therefore, it was necessary to “bundle” the late postoperative day specimens into weeklong blocks (POD 7–13, POD 14–20, POD 21–27, and POD 28–34) and to consider these as single time points.

As regards study B, the wound fluid study, as per the study protocol, in addition to preop blood samples, postop blood, and wound fluid (WF) samples were simultaneously collected at multiple time points (POD1, POD3, POD 7–13, and POD 14–20) from the WF study population.

All blood and wound fluid samples for the two studies were processed within 6 h of collection. Raw WF and blood samples were centrifuged at 16,000*g* for 10 min at 6 °C and aliquots of plasma and WF supernatant collected. Plasma and WF samples were stored at − 80 °C until the assay was performed. Blood samples were collected in heparin-containing tubes.

### CXCL16 determination

Plasma and WF levels of CXCL16 were analyzed in duplicate using commercially available enzyme-linked immunosorbent assay (ELISA) (R&D Systems, Minneapolis, USA) according to the manufacturer’s instructions. CXCL16 concentrations in plasma and WF are reported as nanograms per milliliter (ng/ml).

### Statistical analysis

**Fig. 1:**
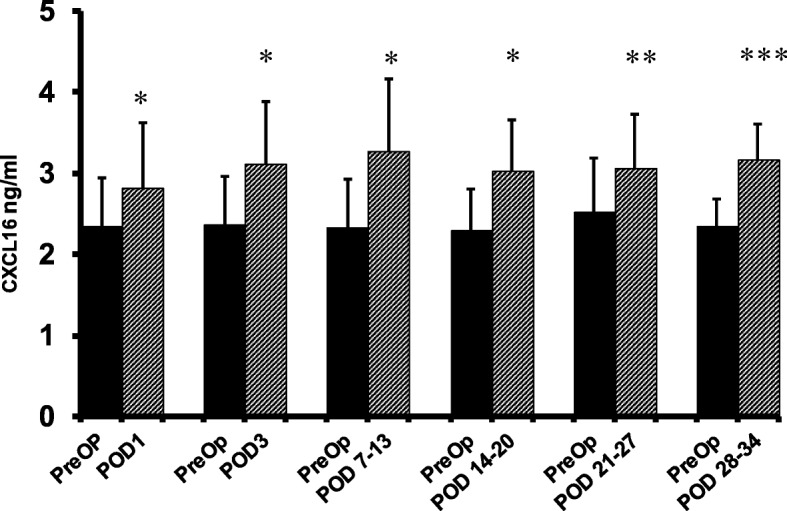
ELISA-determined preoperative (preop) and postoperative plasma CXCL16 levels of colorectal cancer patients. CXCL16 levels are expressed as Mean ± SD. *preop vs. POD 1 (*n* = 86), preop vs. POD 3 (*n* = 82), preop vs. POD 7–13 (*n* = 64); preop vs. POD 14–20 (*n* = 24) *p* = < 0.0001; **preop vs. POD 21–27 (*n* = 20) *p* = 0.0003; ***POD 28–34 time point (*n =* 11, *p* = 0.001)

**Fig. 2:**
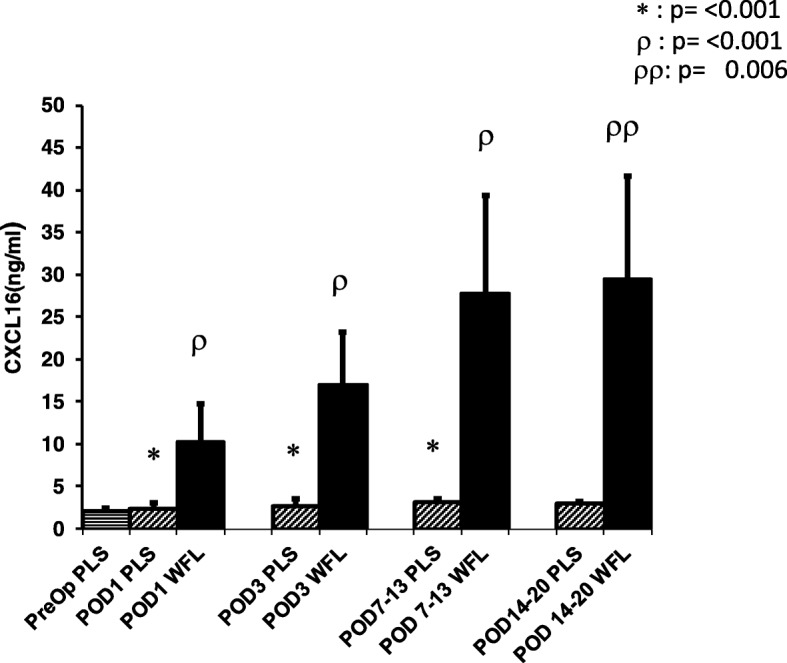
ELISA-determined preoperative (preop) and postoperative CXCL16 levels of plasma and wound fluid levels of colorectal cancer patients. CXCL16 levels are presented as medians with 95% CI *preop vs. POD 1 (*n* = 23), preop vs. POD 3 (*n* = 14), preop vs. POD 7–13(*n* = 10) *p* = < 0.0001; preop vs. POD 14–20 (*n* = 4) ns, plasma vs. wound fluid (WFS); ^ρ^POD1 (plasma, *n* = 23) vs. POD1 (WFS, *n* = 22); POD3 (plasma, *n* = 14) vs. POD3 (WFS, *n* = 17); POD7–13 (plasma, *n* = 10) vs. POD7–13 (WFS, *n* = 15), *p* < 0.0001; ^ρρ^POD14–20 (plasma, n = 4) vs. POD14–20 (WFS, *n* = 7), *p* = 0.006

For preoperative vs. postoperative (postop) plasma CXCL16 comparisons, the data is reported as mean ± standard deviation and the Wilcoxon signed-rank test was used. The results of the postop plasma vs. WF comparisons are stated as the median and 95% confidence interval, and the data was analyzed by the Mann-Whitney *U* test. Spearman’s rank correlation coefficient (rs) was used to evaluate the correlation between postoperative CXCL16 levels vs. incision size, and length of surgery. SPSS version 15.0 (SPSS, Inc., Chicago, IL) was used for data analysis. A separate preoperative (preop) result bar is included for each postoperative (postop) time point in Figs. 1 and 2, as the sample size varies among postoperative time points.

## Results

### Study A

Preoperative and one or more late postoperative plasma sample were available for 86 CRC patients who underwent MICR (colon 73%; rectal 27%; 40 male/46 female, mean age 64.8 ± 12.9 years) for plasma CXCL16 study. The majority of patients underwent laparoscopic-assisted (lap) procedures (63%) while the remainder had hand-assisted laparoscopic resections. The mean incision length was 7.4 ± 4.3 cm (lap) and 10.2 ± 3.9 cm (hand-assisted procedure). The mean operative time was 321.8 ± 119.7 min. The mean length of stay was 6.5 ± 3.9 days (Table 1). The final pathological stage breakdown was as follows: stage I, 30%; stage II, 29%; stage III, 37%; and stage IV, 4%. The majority of patients underwent right colectomy (32%) followed by sigmoid (25%) and LAR/AR resection (22%). Seven (7) complications (8.0%) were noted, including wound infection (1), hematoma (1), hyperkalemia (1), hernia (1), and small bowel obstruction (3).

**Table 1:** Demographic and clinical characteristics of the plasma CXCL16 study population (study A)

	Cancer (*n* = 86)
Age, years (mean ± SD)	64.8 ± 12.93
Sex (*n*)	
Male	40 (46.5%)
Female	46 (53.5%)
Incision length, cm (mean ± SD)	8.4 ± 4.3
Operative time, min (mean ± SD)	321.8 ± 119.7
Length of stay, days (mean ± SD)	6.5 ± 3.9
Type of resection	
Right	26 (32.0%)
Transverse	5 (5.0%)
Left	6 (7.0%)
Sigmoid/rectosigmoid	12 (25.0%)
LAR/AR	27 (22.0%)
APR	3 (2.0%)
Subtotal/total	7 (7.0%)
Surgical method	
Laparoscopic-assisted (LA)	54(63.0%)
Hand-assisted/hybrid laparoscopic (HAL)	32(37.0%)

The mean preoperative CXCL16 level was 2.36 ± 0.57 ng/ml (*n* = 86). Significantly elevated mean plasma levels were noted on POD1 (2.82 ± 0.81, *n* = 86, *p* <  0.0001), POD3 (3.12 ± 0.77, *n* = 82, *p* <  0.0001), POD7–13 (3.28 ± 0.88, *n* = 64, *p* <  0.0001), POD14–20 (3.03 ± 0.62, *n* = 24, *p* <  0.0001), POD 21–27 (3.06 ± 0.67, *n* = 20, *p* = 0.0003), and POD 28–34 (3.17 ± 0.43, *n* = 11, *p* = 0.001) as compared to preoperative levels (Fig. 1). The percent increase in mean plasma CXCL16 levels from baseline were 20% at POD1, 32% at POD3, 40% at POD7–13, 32% at POD14–20, 22.0% at POD21–27, and 35% at POD 28–34. Postoperative plasma CXCL16 levels in the laparoscopic-assisted group were compared to the hand-assisted group to assess for any correlation with incision length. The mean length of incision for the hand-assisted group was greater. There was no statistically significant difference in postoperative plasma CXCL16 levels between the groups. There was no correlation between incision length and plasma CXCL16 level. Furthermore, there was no significant correlation between cancer stage and preoperative plasma CXCL16 level.

### Study B

Wound fluid and plasma samples from 23 CRC patients were obtained (colon 4, rectal 19; 14 males, 9 females; mean age 66.6 ± 10.8 years) in order to determine and compare CXCL16 levels. The surgical methods used were laparoscopic, 13 and hand-assisted, 10 (mean incision length: lap 7.8 ± 1.8; hand 10.5 ± 3.7 cm). The mean length of stay was 8.3 ± 7.8 days (Table 2) and there were three complications (12.0%): urinary retention (2), ileus (1), and hematoma (1). The mean postoperative plasma CXCL16 levels were elevated to a similar extent as observed in study A and the differences when compared to the baseline preoperative plasma results were significant for the first three postop time points. There was no significant difference found for the POD 14–20 time point, however, probably because the *n* is quite small (Fig. 2). As regards the WF vs. plasma comparison, WF levels were significantly elevated at all postop time points (POD1, POD3, POD7–13, and POD14–20; Fig. 2; *p* <  0.05 for all comparisons). The mean WF levels were 3–10 times higher (*p* < 0.05 for all comparisons) than the corresponding plasma levels (Table 3).

**Table 2:** Demographic and clinical characteristics of the plasma wound fluid study population

	Cancer (*n* = 23)
Age, years (mean ± SD)	66.6 ± 10.8
Sex (*n*)	
Male	14 (61.0%)
Female	9 (39.0%)
Incision length, cm (mean ± SD)*	9.1 ± 3.0
Operative time, min (mean ± SD)	456.8 ± 105.5
Length of stay, days (mean ± SD)	8.3 ± 7.8
Type of resection	
Transverse	1 (4.0%)
Sigmoid/rectosigmoid	2 (9.0%)
LAR/AR	12 (52.0%)
APR	7 (31.0%)
Total	1 (4.0%)
Surgical method	
Laparoscopic-assisted (LA)	13(57.0%)
Hand-assisted/hybrid laparoscopic (HAL)	10(43.0%)

*Incision length lap 7.8 ± 1.8; hand 10.5 ± 3.7

**Table 3:** Plasma and wound fluid CXCL16 levels of benign group; values reported as median and 95% CI (study B)

	Plasma (*n*)	Wound fluid (*n*)	*p*
PreOp	2.204 (23)		
C I	1.97–2.35		
POD1	2.517 (23)	10.26 (22)	< 0.0001
C I	1.89–2.51	7.17–14.19	
POD3	2.813 (14)	17.27 (17)	< 0.0001
C I	2.28–3.63	13.44–23.15	
POD 7–13	3.253 (10)	24.37 (15)	< 0.0001
C I	2.65–3.62	20.6–36.28	
POD14–20	3.034 (4)	29.49 (7)	0.006
C I	2.39–3.29	10.11–56.5	

## Discussion

Mean plasma levels of CXCL16 were significantly elevated at six postoperative time points after MICR in a population of 86 CRC patients (study A). In a separate smaller population of CRC patients (mostly rectal cancer), similar plasma elevations were noted (study B). The source of CXCL16 that results in the early plasma elevations after MICR is unclear but may be the acute inflammatory response since CXCL16 has been shown to be upregulated by early inflammatory mediators such as tumor necrosis factor-alpha and interleukin-1 beta [39]. The results of study B suggest that the healing wounds may be a source of CXCL16, especially during weeks 2–3 after surgery. WF levels were 8 to 10 times higher than the comparable plasma levels during the POD 7–13 and POD 14–10 time points after MICR. In light of the evidence that CXCL16 plays a role in angiogenesis, it is not surprising that WF levels are elevated since angiogenesis is integral to wound healing. The fact that wound levels of CXCL16 remain significantly elevated for at least 3 weeks after surgery strongly suggests that there is considerable angiogenesis occurring in the wound throughout this time period. The authors hypothesize that CXCL16 diffuses from the wound into the bloodstream such that plasma levels are elevated.

As mentioned, CXCL16, among other actions, has been shown to play a role in several early steps in the process of angiogenesis as regards EC precursor cell recruitment and the initiation of EC invasion and migration via actions at the epithelial-mesenchymal border such as impairment of E-cadherin, production and induction of vimentin (an intermediate filament protein), and induction of the ERK cell signaling pathway [35, 36, 40, 41]. Thus, CXCL16 is added to the growing list of proteins that play a role in angiogenesis whose plasma levels are elevated for 3 to 5 weeks after colorectal resection. The proteins in question are ANG-2, sVCAM-1, VEGF, MCP-1, PlGF, MMP-2, MMP-3, CHi3L1 (chitinase-3-like protein-1), KGF (keratinocyte growth factor), osteopontin (OPN), progranulin, and IL-8 [17–22, 42]. As mentioned, there is in vitro EC culture data suggesting that the plasma from the second and third weeks after surgery stimulates EC proliferation, invasion, and migration. All of which are critical to the process of neovascularization [17, 24, 25]. What are the possible clinical implications of these plasma compositional changes?

It is feared that the proangiogenic plasma may promote angiogenesis in residual tumor deposits that are present in a subpopulation of CRC patients undergoing “curative” resection during the first month after surgery. There is no definitive evidence directly linking these changes in plasma composition to early recurrence or accelerated tumor growth after surgery. However, there is at least one study which demonstrated that primary tumor resection in CRC is associated with an increase in the peri- and intra-tumoral vascular density of synchronous liver metastases 6–12 weeks after surgery [6]. Other studies, concerning CRC patients with synchronous liver lesions that underwent primary tumor resection, that carried out serial CT or PET scans also noted rapid growth of the liver lesions soon after surgery [43–46]. Further study is clearly required to determine if the persistent plasma protein changes noted above are clinically relevant; however, if it is determined that surgery is clearly associated with stimulated tumor growth, then the development of anti-cancer treatments for the perioperative time period would be warranted. This will be a challenging task since any drug or agent used in this time window must be capable of inhibiting tumor growth while not impairing wound healing.

In the present study, no correlation was found between the preoperative plasma CXCL16 levels and the final cancer stage; however, other investigators have noted a direct relationship between blood levels and cancer stage [39]. Matsushita et al. reported that elevated CXCL16 levels were associated with disease progression and poor prognosis [39]. Thus, based on the prior literature, CXCL16 may hold some promise as a tumor marker. Weaknesses of this study include the relatively small number of late plasma samples, the need to bundle the late samples into 7-day time blocks, and the fact that there are no wound fluid samples from beyond the third week. The logistical impossibility of scheduling outpatient follow-up visits on a particular postoperative day as well as the fact that most patients returned to the office only once during the first 3 weeks after surgery are the reasons for the lower “*n*”s for the late time points and the need to bundle the specimens. There was no late wound fluid because by the fourth week after surgery, the intrabdominal drains had been removed.

## Conclusion

MIS colorectal resection in CRC patients is associated with significant elevations in plasma CXCL16 levels for a full month after surgery. Furthermore, the levels of CXCL16 in “wound” fluid from the site of surgery in the pelvis were found to be 3 to 10 times higher than the corresponding plasma levels for the first 3 weeks after colorectal surgery. These results suggest that the wound may be the source of the added protein late after surgery. CXCR16 enters to the group of proteins with proangiogenic effects whose blood levels are persistently elevated after colorectal resection. Further study is warranted both to determine the impact of these surgery-related changes and to better understand the effects of surgery.

## Abbreviations

CRC: Colorectal cancer
IRB: Institutional Review Board
MICR: Minimally invasive colorectal resection
POD: Postoperative day
preop: Preoperative
pts: Patients
WF: Wound fluid
